# Carpal Tunnel Syndrome and Other Predictors of Amyloidosis

**DOI:** 10.1016/j.jhsg.2026.100952

**Published:** 2026-02-17

**Authors:** Kathryn E. Grabowski, Stephen C. Vlay, Peter Gorevic, Marie A. Badalamente, David E. Komatsu, Lawrence C. Hurst

**Affiliations:** ∗ Renaissance School of Medicine, Stony Brook University, Stony Brook, NY; † Department of Cardiology, Stony Brook University, NY; ‡ Division of Rheumatology, Allergy and Immunology, Stony Brook University, Health Sciences Center, Stony Brook, NY; § Department of Orthopaedics and Rehabilitation, Stony Brook University, Health Sciences Center, Stony Brook, NY

**Keywords:** Amyloidosis, Cardiac amyloidosis, Carpal tunnel syndrome, Transthyretin

## Abstract

**Purpose:**

Amyloidosis comprises a broad category of pathologies characterized by extracellular deposits of amyloid. Deposition in the myocardium causes cardiac amyloidosis, a progressive and frequently fatal disease. Recent studies have reported that elderly patients with carpal tunnel syndrome (CTS) may have amyloid deposits in their flexor tenosynovium at the time of carpal tunnel release. However, the overall risk of these patients having amyloidosis is low, and there is a need to identify additional risk factors to guide surgeons in deciding which patients should undergo biopsies. This study sought to identify and rank risk factors based on their impact on increasing amyloidosis rates among carpal tunnel patients.

**Methods:**

We conducted a retrospective cohort study of patients with CTS between 2010 and 2019 using the TriNetX Research Network to determine the incidence of amyloidosis within 6 years following a CTS diagnosis and identify preexisting risk factors. Men and women were analyzed separately, and all risk factors were examined in yearly increments from 1 to 6 years after CTS diagnosis and risk differences were reported.

**Results:**

Of the 30 hypothesized risk factors, 18 were notable for men aged ≥50 and 19 were notable for women aged ≥50, each increasing the likelihood of developing amyloidosis within 6 years of a CTS diagnosis. The strongest risk factor for both sexes was heart failure, though the absolute risk difference was lower in women than in men, with an increased risk of 261 per 100,000 compared to 702 per 100,000.

**Conclusions:**

For both men and women with CTS, there are several preexisting conditions associated with an increased risk of developing amyloidosis within six years of a CTS diagnosis. *Clinical relevance*: These additional risk factors may identify high-risk patients that should be recommended for a tenosynovial biopsy at the time of their CTS surgical release.

Amyloidosis is a protein misfolding disease caused by extracellular deposits of amyloid fibrils.^1^, ^2^, ^3^ While 42 specific forms have been identified, two main types of amyloidosis, amyloid transthyretin (ATTR) and immunoglobulin or amyloid light chain (AL), account for 95% of amyloidosis cases.^1^, ^2^, ^3^, ^4^ ATTR has two subtypes: wild type (ATTRwt) and variant or hereditary type (ATTRv).^2^ ATTR and AL are the two main types that deposit amyloid in the myocardium, leading to cardiac amyloidosis.^5^

The prevalence of ATTRv is unknown, as it is inherited as an autosomal dominant disease with variable penetrance.^1^, ^2^, ^3^ Without taking into account the general population, one study estimated the prevalence of ATTRwt to be approximately 1:6,000 among a cohort of heart failure patients.^3^ Amyloidosis secondary to AL is a rare disease with an incidence of 15–40 per million in the United States, with 12,000 currently living with amyloidosis.^2^^,^^3^ Despite the lack of estimates for the prevalence of ATTR, it is generally thought to be more common, though less severe than AL.^2^, ^3^, ^4^

Recent studies have shown that some patients with carpal tunnel syndrome (CTS), specifically men ≥50 and women ≥60, have amyloid deposits in their flexor tenosynovium at the time of carpal tunnel release.^2^^,^^3^^,^^6^ Importantly, these patients did not demonstrate any overt signs of systemic or cardiac amyloidosis. However, follow-up testing revealed that the presence of amyloid deposits predicted the development of debilitating and potentially fatal amyloidosis problems involving the major organs, such as the heart, 5–10 years later.^2^^,^^3^^,^^7^, ^8^, ^9^ Specifically, Sood and Lipira^6^ reported that patients’ CTS diagnosis preceded an amyloidosis diagnosis by a median of 5.1 years. Unfortunately, the number of patients included in these recent studies of CTS and amyloidosis has been relatively small. In 29 recent studies, the largest cohort of cardiac amyloidosis patients studied was 1,310.^1^, ^2^, ^3^^,^^6^, ^7^, ^8^, ^9^, ^10^, ^11^

Based on these small studies, the current “red flags” that suggest a patient with CTS might have an increased risk for a positive biopsy for amyloid include: advanced age, male sex, African American race, thyroid disease, heart disease, lumbar stenosis, rotator cuff tear, biceps rupture, trigger finger, irritable bowel syndrome, weight loss, back or neck pain, diabetes, neuropathy, eye problems, enlarged tongue, history of joint replacement, or sleep apnea. These “red flags” have been tiered, but the strength of the associations between these comorbidities and amyloidosis has not been ranked.^5^^,^^12^ If these asymptomatic amyloid patients with carpal tunnel syndrome can be identified early, the recently developed new class of amyloidosis drugs can potentially slow the deposition of amyloid in major organs and improve morbidity and mortality.^1^, ^2^, ^3^^,^^13^, ^14^, ^15^ Therefore, identifying which patients could benefit from biopsies during CTS surgical release because of their amyloidosis risk is important and potentially lifesaving. In this study, the TriNetX database was used to assess the relative predictability of each “red flag” so that the risk of a patient with CTS having undiagnosed amyloidosis could be more accurately assessed prior to recommending a tenosynovial biopsy at the time of carpal tunnel release.

## Materials and Methods

This was a retrospective cohort study of patients with CTS, amyloidosis, and 30 “red flag” diagnoses.^1^^,^^2^^,^^4^^,^^5^^,^^12^^,^^16^, ^17^, ^18^, ^19^, ^20^, ^21^ The data were collected from the TriNetX Research Network, which provides access to electronic medical records from approximately 144 million patients across 103 health care organizations. All data displayed on the TriNetX platform in aggregate form, or any patient-level data provided in a data set generated by the TriNetX platform, only contains deidentified data. Because this study used only deidentified patient records and did not involve the collection, use, or transmittal of individually identifiable data, this study was exempted from Institutional Review Board approval. Further information regarding TriNetX’s data can be found at the following website: https://support.trinetx.com/hc/en-us/sections/360000928753-About-the-Data.

The study included electronic medical records data from patients included in the TriNetX Research Network as of September 23, 2025. All cohorts were composed of patients with the given diagnoses between January 1, 2010, and December 31, 2019. We used Current Procedural Terminology (CPT) and International Classification of Diseases, 10th edition (ICD-10), to identify patients with the diagnoses of interest.

**Figure 1:**
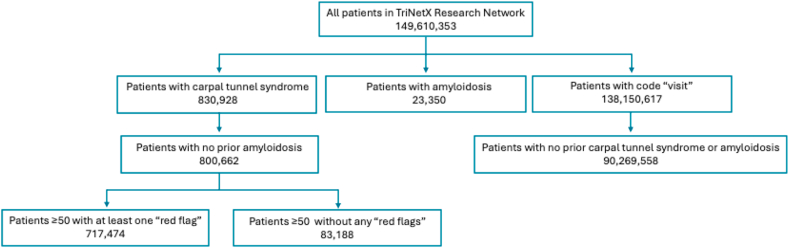
Enrollment flow chart. This flow chart presents the number total number of patients in the TriNetX database, followed by those with CTS, amyloidosis, and at least one recorded visit. From those with a visit, those with no history of CTS or amyloidosis were used as a control group. The cohort with carpal tunnel was then restricted to patients without amyloidosis. To assess risk factors for the development of amyloidosis, potential “red flags” were assessed in patients ≥50.

Three primary cohorts were defined for the analysis: a CTS cohort, a general population cohort, and an amyloidosis cohort. The CTS cohort included patients with a diagnosis code of CTS (G56.0), but no prior diagnosis of amyloidosis (E85.1, E85.4, E85.81, E85.82). The general population cohort included patients with a code for a physician visit, but no prior diagnosis of amyloidosis (E85.1, E85.4, E85.81, E85.82) or CTS (G56.0). The amyloidosis cohort included patients with neuropathic heredofamilial amyloidosis (E85.1), organ-limited amyloidosis (E85.4), light chain amyloidosis (E85.81), or wild type transthyretin-related amyloidosis (E85.82). The number of patients in each of these cohorts is presented in the Figure 1.

To assess preexisting risk among patients aged ≥50, additional analyses were performed within the CTS cohort.^2^^,^^3^^,^^6^^,^^22^ For these analyses, all patients had a diagnosis of CTS (G56.0) and no prior diagnosis of amyloidosis (E85.1, E85.4, E85.81, or E85.82). For each “red flag” diagnosis, two cohorts were made: one cohort consisting of CTS patients with the “red flag” diagnosis documented prior to CTS and a comparator cohort consisting of CTS patients without that “red flag” diagnosis prior to CTS. This approach resulted in a total of 60 red flag-specific cohorts. The following preexisting risk factors were examined: African American race, heart failure (I50), trigger finger (M65.3), diabetes mellitus type 2 (E11), atrial fibrillation (AFib) or atrial flutter (I48.1 I48.11, I48.2, I48.0, I48.3 or I48.4), aortic stenosis (AS) (I35), cardiomyopathy or chest pain (I43 or R07.8), open CTS surgical release (64721), endoscopic CTS surgical release (29848), peripheral neuropathy (G60.9 or G62.9), lumbar spinal stenosis (LSS) (M48.06), total knee arthroplasty (27447), total hip arthroplasty (THA) (27130), rotator cuff tear (M75.1), family history of heart disease (Z82.49), sleep apnea (G47.3), noncentral causes of dizziness (I95.1, H81.39 or R42), essential hypertension (I10), bilateral CTS (G56.03), shortness of breath (R06.02), localized edema (R60.0), low back pain (M54.5), diabetes mellitus type 1 (E10), digestive issues or abnormal weight loss (K00-K95 or R63.4), floaters (H43.39), enlarged tongue (K14.8), diabetic neuropathy (E11.43, E11.40, E11.41, or E11.42), Dupuytren contracture (M72), distal biceps tendon rupture (M66.821, M66.822, M66.829, M66.82, S46.1, 24342, 23440, or 23430), and presence of a cardiac pacemaker (Z95.0).^1^^,^^2^^,^^4^^,^^5^^,^^12^^,^^16^, ^17^, ^18^, ^19^, ^20^, ^21^ The number of patients aged ≥50 with at least one or none of these risk factors is presented in the Figure 1.

Patients were followed in yearly intervals from 1 to 6 years after the index diagnosis of CTS to assess the incidence of amyloidosis, with analyses stratified by sex (male or female, as reported in TriNetX). First, patients with CTS were compared with the general population to evaluate the overall risk of developing amyloidosis associated with CTS. Second, within the CTS cohort, patients with each of the 30 predefined “red flag” diagnoses were compared with CTS patients without that specific red flag diagnosis. This resulted in a total of 31 cohort comparisons. For example, the first-time interval is from 1 day to 1 year after the index event, then 1 year to 2 years after the index event, etc, up to year 6. We used the TriNetX “Compare Cohorts” tool to determine measures of association. Comparisons were performed after 1:1 matching on age at the index event to create age-balanced cohorts of equal size. The index event was the diagnosis of CTS for most cohorts, except the general population cohort, in which case the index event was the clinic visit. For the cohorts with preexisting risk factors, the tabular results were ordered from strongest to weakest association based on the lowest *P* values and the graphical results were ordered from strongest to weakest based on risk difference. For the graphical results, the risk of developing amyloidosis in the CTS cohort without the additional risk factor was subtracted from the risk in the CTS cohort with the additional risk factor to yield the risk difference. The risk difference was then scaled to reflect a population of 100,000. We highlighted cohorts with <5,000 patients because they tend to be too small a population to find considerable differences in, since TriNetX will not report a specific numeric population for any outcome with ≤10 patients.

## Results

**Figure 2:**
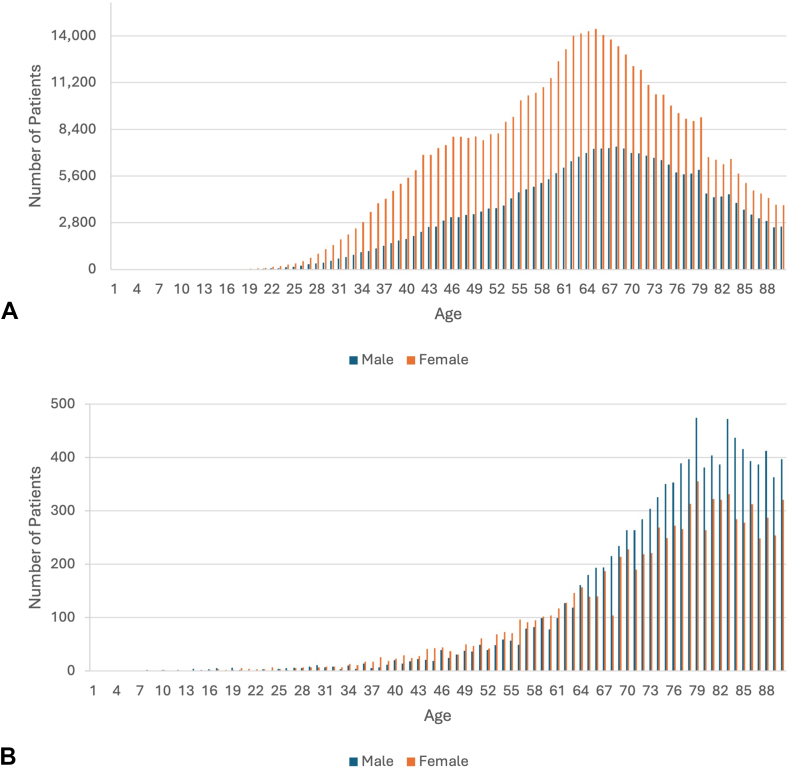
Age distribution of cohorts. **A** Patients with CTS separated by sex. A total of 756,618 patients is shown on this graph, with 44,044 patients aged 90 and older not plotted. **B** Patients with amyloidosis separated by sex. A total of 19,072 patients is shown on this graph, with 4,278 patients aged 90 and older not plotted.

The baseline demographic data of the CTS and amyloidosis cohorts, prior to any age matching or adjustment, are shown in the Table 1. Notably, the amyloidosis cohort was older than the CTS cohort (76 vs 63). Men were considerably more prevalent in the amyloidosis cohort (54.25%), and women were more prevalent in the CTS cohort (66.10%). Race was similar for both cohorts, with most subjects identified as White, followed by Black, Asian, and Unknown. The distribution of age and sex for patients within the CTS cohort is shown in the Figure 2A. The distribution of age and sex for the amyloidosis cohort is shown in the Figure 2B.

**Table 1:** Demographic Characteristics for CTS and Amyloidosis Cohorts

Total Patients	CTS	Amyloidosis
Total patients	800,662	23,350
Mean age at index (y)	63	76
Sex		
Male	33.90%	54.25%
Female	66.10%	45.75%
Race		
White	68.79%	65.31%
Black	14.56%	17.04%
Asian	3.79%	5.77%
Unknown	8.41%	8.07%

**Figure 3:**
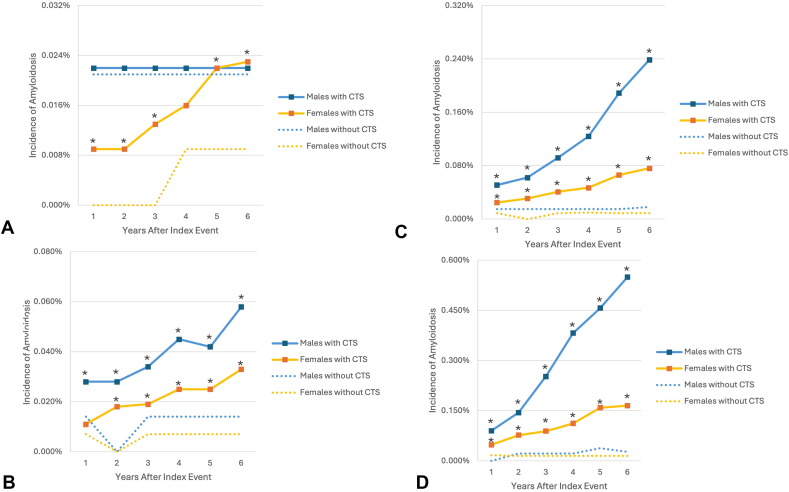
Development of amyloidosis over 6 years following the index event, stratified by sex. **A** In patients aged 40–49 with and without CTS. Among men, incidence was identical with and without CTS (0.022% in all years; *P* > .99), so the non-CTS line is shown slightly offset for clarity. However, women showed significant differences in the development of amyloidosis in all years, except year 4, as shown by the asterisks (∗) (*P* = .002, .002, <.001, .178, .016, .011). **B** In patients aged 50–59 with and without CTS. There are 16 significant differences in the development of amyloidosis in men across all 6 years (*P* = .048, <.001, .011, <.001, .001, <.001). Women show significant differences in the development of amyloidosis in all years, except year 1 (*P* = .118, <.001, .002, <.001, <.001, <.001). Statistically significant values are shown by the asterisks (∗). **C** In patients aged 60–69 with and without CTS. There are significant differences in the development of amyloidosis in both men (*P* < .001, <.001, <.001, <.001, <.001, <.001) and women (*P* = .005, <.001, <.001, <.001, <.001, <.001) across all 6 years, as shown by the asterisks (∗). **D** In patients aged ≥70 with and without CTS. There are significant differences in the development of amyloidosis in both men (*P* < .001, <.001, <.001, <.001, <.001, <.001) and women (*P* = .002, <.001, <.001, <.001, <.001, <.001) across all 6 years, as shown by the asterisks (∗).

For the 40–49-year-olds, CTS did not confer a risk of amyloidosis in men but did for women in every year except year 4 (Fig. 3A). For the 50–59-year-olds, CTS did confer a risk of amyloidosis in men in all 6 years, and in women, all years except year 1 (Fig. 3B). For the 60–69-year-olds and the ≥70-year-olds, CTS did confer a risk of amyloidosis in all 6 years for men and women (Fig. 3C, D).

Comparisons were then made separately for men and women aged ≥50 to determine which preexisting conditions/characteristics, prior to a CTS diagnosis, further increase the risk of developing amyloidosis. For the men aged ≥50 and the women aged ≥50, the risk of developing amyloidosis was assessed in 1-year intervals over the first 6 years and cumulatively in the 6 years following the CTS diagnosis (Tables 2 and 3). For both the Tables 2 and 3, the preexisting conditions are listed from strongest to weakest strength of association based on the frequency of statistically significant risks in the six years following the index event. The risk factors that were found to be statistically insignificant (*P* > .05) and/or had cohort populations <5,000 are shown in the Table 4 for men and the Table 5 for women.

**Table 2:** Significant Factors for Developing Amyloidosis 312 During Years 1–6 Following CTS Diagnosis in Patients Aged ≥50, With and Without an Additional Risk Factor Present Prior to CTS Diagnosis in Men

Risk Factor	Y After Index Event	Risk in CTS Cohort With Additional Risk Factor	Risk in CTS Cohort Without Additional Risk Factor	*P* Value
Shortness of breath	1	0.168%	≤0.042%	**<.001**
	2	0.205%	≤0.042%	**<.001**
	3	0.226%	0.071%	**<.001**
	4	0.272%	0.130%	**<.001**
	5	0.360%	0.176%	**<.001**
	6	0.402%	0.230%	**<.001**
	Total	0.799%	0.445%	**<.001**
Heart failure	1	0.294%	0.126%	**.005**
	2	0.310%	0.101%	**<.001**
	3	0.428%	0.201%	**.002**
	4	0.503%	0.277%	**.005**
	5	0.528%	0.327%	**.017**
	6	0.562%	0.352%	**.016**
	Total	1.223%	0.522%	**<.001**
Cardiomyopathy or chest pain	1	0.116%	≤0.043%	**.005**
	2	0.129%	≤0.043%	**.002**
	3	0.172%	0.073%	**.002**
	4	0.211%	0.120%	**.017**
	5	0.288%	0.181%	**.017**
	6	0.361%	0.241%	**.018**
	Total	0.611%	0.330%	**<.001**
Bilateral CTS	1	0.089%	0.029%	**<.001**
	2	0.093%	0.064%	.108
	3	0.176%	0.089%	**<.001**
	4	0.234%	0.145%	**.002**
	5	0.333%	0.159%	**<.001**
	6	0.393%	0.209%	**<.001**
	Total	0.651%	0.372%	**<.001**
Lumbar spinal stenosis	1	0.111%	≤0.062%	.130
	2	0.167%	≤0.062%	**.005**
	3	0.322%	0.093%	**<.001**
	4	0.353%	0.130%	**<.001**
	5	0.470%	0.210%	**<.001**
	6	0.509%	0.285%	**.001**
	Total	0.986%	0.503%	**<.001**
African American	1	0.101%	≤0.056%	.130
	2	0.112%	≤0.056%	.068
	3	0.201%	0.067%	**<.001**
	4	0.274%	0.084%	**<.001**
	5	0.296%	0.117%	**<.001**
	6	0.394%	0.168%	**<.001**
	Total	0.671%	0.345%	**<.001**
Noncentral causes of dizziness	1	0.103%	0.069%	.206
	2	0.176%	0.077%	**.003**
	3	0.223%	0.158%	.112
	4	0.300%	0.188%	**.015**
	5	0.360%	0.253%	**.036**
	6	0.377%	0.257%	**.021**
	Total	0.739%	0.398%	**<.001**
Atrial fibrillation or atrial flutter	1	≤0.143%	≤0.143%	>.999
	2	0.258%	0.243%	.866
	3	0.415%	0.243%	.076
	4	0.529%	0.300%	**.035**
	5	0.601%	0.286%	**.005**
	6	0.758%	0.286%	**<.001**
	Total	1.373%	0.687%	**<.001**
Essential hypertension	1	0.052%	0.046%	.637
	2	0.075%	0.048%	**.035**
	3	0.104%	0.063%	**.007**
	4	0.160%	0.104%	**.003**
	5	0.180%	0.147%	.121
	6	0.226%	0.162%	**.005**
	Total	0.399%	0.277%	**<.001**
Peripheral neuropathy	1	0.146%	0.042%	**<.001**
	2	0.149%	0.107%	.179
	3	0.188%	0.115%	**.032**
	4	0.281%	0.169%	.195
	5	0.313%	0.176%	**.002**
	6	0.373%	0.236%	**.006**
	Total	0.735%	0.463%	**<.001**
Trigger finger	1	0.063%	0.094%	.317
	2	0.100%	≤0.063%	.239
	3	0.188%	0.113%	.083
	4	0.288%	0.150%	**.009**
	5	0.389%	0.194%	**.001**
	6	0.545%	0.244%	**<.001**
	Total	0.791%	0.503%	**.002**
Aortic stenosis	1	0.197%	0.152%	.531
	2	0.228%	0.167%	.432
	3	0.402%	0.232%	.085
	4	0.531%	0.243%	**.008**
	5	0.622%	0.258%	**.002**
	6	0.652%	0.334%	**.009**
	Total	1.276%	0.816%	**.014**
Sleep apnea	1	0.056%	0.077%	.297
	2	0.086%	0.070%	.686
	3	0.145%	0.098%	.071
	4	0.230%	0.145%	**.010**
	5	0.281%	0.177%	**.005**
	6	0.352%	0.216%	**<.001**
	Total	0.571%	0.397%	**.001**
Open CTS surgical release	1	≤0.039%	0.059%	.317
	2	0.063%	0.063%	>.999
	3	0.138%	0.071%	**.020**
	4	0.217%	0.134%	**.026**
	5	0.280%	0.189%	**.035**
	6	0.335%	0.256%	.102
	Total	0.528%	0.336%	**.001**
Localized edema	1	0.118%	≤0.069%	.178
	2	0.152%	0.131%	.639
	3	0.194%	0.180%	.785
	4	0.353%	0.207%	**.020**
	5	0.394%	0.235%	**.016**
	6	0.394%	0.311%	.234
	Total	0.817%	0.515%	**.002**
Family history of heart disease	1	0.173%	≤0.133%	.531
	2	0.199%	0.146%	.432
	3	0.226%	≤0.133%	.178
	4	0.332%	0.159%	**.032**
	5	0.411%	0.146%	**.002**
	6	0.385%	0.239%	.108
	Total	0.838%	0.249%	**<.001**
Low back pain	1	0.045%	0.067%	.189
	2	0.090%	0.057%	.075
	3	0.167%	0.098%	**.006**
	4	0.186%	0.145%	.149
	5	0.243%	0.207%	.275
	6	0.278%	0.238%	.248
	Total	0.539%	0.361%	**<.001**
Digestive issues or abnormal weight loss	1	0.056%	0.047%	.448
	2	0.081%	0.058%	.076
	3	0.114%	0.083%	.050
	4	0.151%	0.132%	.295
	5	0.196%	0.170%	.227
	6	0.242%	0.198%	.059
	Total	0.448%	0.315%	**<.001**
Rotator cuff tear	1	≤0.065%	≤0.065%	>.999
	2	0.098%	0.085%	.705
	3	0.157%	0.111%	.274
	4	0.183%	0.150%	.484
	5	0.294%	0.170%	**.024**
	6	0.307%	0.228%	.185
	Total	0.523%	0.430%	.242

Risk factors are ordered from strongest to weakest association with amyloidosis risk. Bold *P* values indicate statistical significance with *P* < .05.

**Table 3:** Significant Factors for Developing Amyloidosis 312 During Years 1–6 Following CTS Diagnosis in Patients Aged ≥50, With and Without an Additional Risk Factor Present Prior to CTS Diagnosis in Women

Risk Factor	Y After Index Event	Risk in CTS Cohort With Additional Risk Factor	Risk in CTS Cohort Without Additional Risk Factor	*P* Value
Peripheral neuropathy	1	0.069%	≤0.029%	**.016**
	2	0.069%	≤0.029%	**.016**
	3	0.107%	0.032%	**<.001**
	4	0.107%	0.043%	**.002**
	5	0.118%	0.072%	**.049**
	6	0.162%	0.081%	**.002**
	Total	0.374%	0.177%	**<.001**
African American	1	0.039%	≤0.019%	.068
	2	0.072%	0.023%	**<.001**
	3	0.080%	0.027%	**<.001**
	4	0.092%	0.023%	**<.001**
	5	0.119%	0.021%	**<.001**
	6	0.160%	0.049%	**<.001**
	Total	0.356%	0.120%	**<.001**
Heart failure	1	0.110%	≤0.061%	.130
	2	0.147%	≤0.061%	**.016**
	3	0.141%	≤0.061%	**.024**
	4	0.153%	≤0.061%	**.011**
	5	0.153%	0.067%	**.020**
	6	0.165%	0.092%	**.064**
	Total	0.483%	0.222%	**>.001**
Essential hypertension	1	0.026%	0.016%	.074
	2	0.040%	0.025%	**.035**
	3	0.049%	0.026%	**.003**
	4	0.061%	0.035%	**.004**
	5	0.068%	0.063%	.122
	6	0.081%	0.060%	**.050**
	Total	0.188%	0.115%	**<.001**
Bilateral CTS	1	0.034%	0.021%	.101
	2	0.053%	0.035%	.079
	3	0.052%	0.040%	.251
	4	0.067%	0.052%	.225
	5	0.103%	0.057%	**>.001**
	6	0.129%	0.057%	**<.001**
	Total	0.266%	0.156%	**<.001**
Shortness of breath	1	0.031%	≤0.022%	.414
	2	0.066%	0.040%	.083
	3	0.083%	0.037%	**.005**
	4	0.092%	0.057%	.052
	5	0.114%	0.081%	.112
	6	0.143%	0.097%	**.044**
	Total	0.335%	0.155%	**<.001**
Digestive issues or abnormal weight loss	1	0.022%	0.019%	.593
	2	0.033%	0.028%	.442
	3	0.045%	0.026%	**.008**
	4	0.050%	0.034%	.050
	5	0.065%	0.054%	.209
	6	0.078%	0.058%	**.047**
	Total	0.191%	0.108%	**<.001**
Trigger finger	1	0.028%	≤0.028%	>.999
	2	0.042%	0.048%	.723
	3	0.042%	0.050%	.601
	4	0.084%	0.042%	**.025**
	5	0.106%	0.067%	.075
	6	0.154%	0.084%	**.007**
	Total	0.264%	0.162%	**.004**
Low back pain	1	0.026%	0.016%	.170
	2	0.038%	0.028%	.276
	3	0.058%	0.026%	**.002**
	4	0.058%	0.054%	.753
	5	0.087%	0.066%	.128
	6	0.102%	0.075%	.067
	Total	0.234%	0.154%	**<.001**
Lumbar spinal stenosis	1	0.066%	≤0.041%	.239
	2	0.046%	0.046%	>.999
	3	0.090%	0.057%	.182
	4	0.106%	0.061%	.086
	5	0.163%	0.086%	**.015**
	6	0.114%	0.118%	.895
	Total	0.373%	0.214%	**.001**
Cardiomyopathy or chest pain	1	0.032%	0.027%	.705
	2	0.050%	0.032%	.150
	3	0.067%	0.038%	**.048**
	4	0.074%	0.055%	.249
	5	0.067%	0.095%	.138
	6	0.118%	0.086%	.128
	Total	0.283%	0.154%	**<.001**
Noncentral causes of dizziness	1	0.022%	0.027%	.549
	2	0.055%	0.029%	**.047**
	3	0.065%	0.045%	.181
	4	0.078%	0.057%	.185
	5	0.088%	0.082%	.748
	6	0.121%	0.094%	.181
	Total	0.277%	0.175%	**<.001**
Diabetic neuropathy	1	≤0.051%	≤0.051%	>.999
	2	0.057%	≤0.051%	.827
	3	0.098%	≤0.051%	.095
	4	0.098%	≤0.051%	.095
	5	0.108%	≤0.051%	**.048**
	6	0.123%	0.072%	.105
	Total	0.360%	0.191%	**.002**
Diabetes mellitus type 2	1	0.031%	0.021%	.262
	2	0.041%	0.039%	.796
	3	0.057%	0.043%	.204
	4	0.067%	0.057%	.468
	5	0.077%	0.087%	.528
	6	0.099%	0.095%	.803
	Total	0.252%	0.168%	**<.001**
Localized edema	1	0.058%	≤0.032%	.131
	2	0.065%	0.045%	.303
	3	0.081%	0.058%	.286
	4	0.084%	0.071%	.564
	5	0.097%	0.094%	.896
	6	0.130%	0.107%	.412
	Total	0.335%	0.205%	**.002**
Sleep apnea	1	0.035%	≤0.023%	.317
	2	0.035%	≤0.023%	.317
	3	0.039%	0.032%	.599
	4	0.053%	0.039%	.343
	5	0.074%	0.078%	.806
	6	0.099%	0.067%	.099
	Total	0.226%	0.138%	**.003**
Open CTS surgical release	1	≤0.026%	0.034%	.532
	2	0.060%	0.039%	.194
	3	0.080%	0.057%	.216
	4	0.057%	0.052%	.758
	5	0.091%	0.078%	.535
	6	0.124%	0.104%	.394
	Total	0.240%	0.152%	**.007**
Diabetes mellitus type 1	1	≤0.085%	≤0.085%	>.999
	2	≤0.085%	≤0.085%	>.999
	3	≤0.085%	≤0.085%	>.999
	4	≤0.085%	≤0.085%	>.999
	5	0.102%	≤0.085%	.670
	6	≤0.085%	≤0.085%	>.999
	Total	0.266%	0.133%	**.025**
Family history of heart disease	1	≤0.072%	≤0.072%	>.999
	2	≤0.072%	≤0.072%	>.999
	3	≤0.072%	≤0.072%	>.999
	4	0.087%	≤0.072%	.670
	5	0.079%	≤0.072%	.827
	6	0.101%	0.116%	.715
	Total	0.301%	0.172%	**.027**

Risk factors are ordered from strongest to weakest association with amyloidosis risk. Bold *P* values indicate statistical significance with *P* < .05.

**Table 4:** Nonsignificant factors for developing amyloidosis during years 1–6 following CTS diagnosis in patients aged ≥50, with and without an additional risk factor present prior to CTS diagnosis in men

Nonrisk Factor	Y After Index Event	Risk in CTS Cohort With Additional Risk Factor	Risk in CTS Cohort Without Additional Risk Factor	*P* Value
Diabetes mellitus type 2	1	0.052%	0.048%	.768
	2	0.078%	0.059%	.257
	3	0.104%	0.115%	.619
	4	0.131%	0.176%	.077
	5	0.172%	0.229%	.055
	6	0.244%	0.242%	.947
	Total	0.309%	0.360%	.183
Diabetic neuropathy	1	0.081%	≤0.074%	.827
	2	0.088%	0.096%	.841
	3	0.118%	0.162%	.330
	4	0.184%	0.192%	.889
	5	0.162%	0.265%	.066
	6	0.258%	0.309%	.424
	Total	0.486%	0.501%	.862
Diabetes mellitus type 1	1	≤0.131%	≤0.131%	>.999
	2	≤0.131%	≤0.131%	>.999
	3	≤0.131%	0.171%	.531
	4	≤0.131%	0.210%	.239
	5	0.145%	0.237%	.193
	6	0.197%	0.263%	.398
	Total	0.412%	0.465%	.622
Endoscopic CTS surgical release	1	≤0.176%	≤0.176%	>.999
	2	≤0.176%	≤0.176%	>.999
	3	≤0.176%	≤0.176%	>.999
	4	0.264%	≤0.176%	.317
	5	0.300%	0.247%	.590
	6	0.440%	0.229%	.051
	Total	0.688%	0.476%	.139
▲Distal biceps tendon rupture	1	≤0.251%	≤0.251%	>.999
	2	≤0.251%	≤0.251%	>.999
	3	≤0.251%	≤0.251%	>.999
	4	0.276%	≤0.251%	.827
	5	0.276%	≤0.251%	.827
	6	0.427%	≤0.251%	.177
	Total	0.628%	0.452%	.284
▲Total hip arthroplasty	1	≤0.632%	≤0.632%	>.999
	2	≤0.632%	≤0.632%	>.999
	3	≤0.632%	≤0.632%	>.999
	4	≤0.632%	0.000%	.002
	5	≤0.632%	≤0.632%	>.999
	6	0.822%	≤0.632%	.530
	Total	1.051%	0.680%	.255
▲Total knee arthroplasty	1	0.000%	≤0.404%	.002
	2	≤0.404%	0.000%	.002
	3	≤0.404%	≤0.404%	>.999
	4	≤0.404%	≤0.404%	>.999
	5	0.485%	≤0.404%	.669
	6	≤0.404%	≤0.404%	>.999
	Total	0.695%	0.532%	.464
▲Floaters	1	≤0.378%	≤0.378%	>.999
	2	≤0.378%	≤0.378%	>.999
	3	≤0.378%	≤0.378%	>.999
	4	≤0.378%	≤0.378%	>.999
	5	0.530%	≤0.378%	.413
	6	0.492%	0.492%	>.999
	Total	0.821%	0.548%	.235
▲Pacemaker	1	≤0.411%	≤0.411%	>.999
	2	≤0.411%	≤0.411%	>.999
	3	≤0.411%	≤0.411%	>.999
	4	≤0.411%	≤0.411%	>.999
	5	≤0.411%	≤0.411%	>.999
	6	≤0.411%	≤0.411%	>.999
	Total	1.027%	0.698%	.215
**▲**Enlarged tongue	1	0.000%	0.000%	-
	2	0.000%	0.000%	-
	3	**≤**1.828%	≤1.828%	>.999
	4	≤1.828%	0.000%	.002
	5	0.000%	≤1.828%	.002
	6	0.000%	0.000%	-
	Total	1.890%	1.890%	>.999
▲Dupuytren contracture	1	≤0.274%	≤0.274%	>.999
	2	≤0.274%	≤0.274%	>.999
	3	≤0.274%	≤0.274%	>.999
	4	0.302%	≤0.274%	.827
	5	0.357%	≤0.274%	.531
	6	0.521%	≤0.274%	.094
	Total	0.769%	0.549%	.247

Risk factors shown did not reach statistical significance (*P* < .05) and/or had cohort populations <5,000, as indicated by a triangle (▲). Risk factors are ordered by descending strength of association.

**Table 5:** Nonsignificant factors for developing amyloidosis during years 1–6 following CTS diagnosis in patients aged ≥50, with and without an additional risk factor present prior to CTS diagnosis in women

Nonrisk Factor	Y After Index Event	Risk in CTS Cohort With Additional Risk Factor	Risk in CTS Cohort Without Additional Risk Factor	*P* Value
Atrial fibrillation or atrial flutter	1	≤0.166%	≤0.166%	>.999
	2	≤0.166%	≤0.166%	>.999
	3	≤0.166%	≤0.166%	>.999
	4	≤0.166%	≤0.166%	>.999
	5	≤0.166%	≤0.166%	>.999
	6	≤0.166%	≤0.166%	>.999
	Total	0.531%	0.315%	.068
Aortic stenosis	1	≤0.121%	≤0.121%	>.999
	2	0.133%	≤0.121%	.827
	3	0.157%	≤0.121%	.531
	4	0.145%	0.133%	.835
	5	0.193%	0.133%	.336
	6	0.217%	0.229%	.869
	Total	0.466%	0.315%	.127
Rotator cuff tear	1	≤0.045%	≤0.045%	>.999
	2	≤0.045%	≤0.045%	>.999
	3	≤0.045%	0.054%	.670
	4	≤0.045%	≤0.045%	>.999
	5	0.081%	0.076%	.866
	6	0.086%	0.082%	.870
	Total	0.221%	0.157%	.122
Floaters	1	≤0.164%	0.000%	.002
	2	≤0.164%	≤0.164%	>.999
	3	≤0.164%	≤0.164%	>.999
	4	≤0.164%	≤0.164%	>.999
	5	≤0.164%	≤0.164%	>.999
	6	≤0.164%	≤0.164%	>.999
	Total	0.210%	0.262%	.563
Endoscopic CTS surgical release	1	≤0.111%	≤0.111%	>.999
	2	≤0.111%	≤0.111%	>.999
	3	≤0.111%	≤0.111%	>.999
	4	≤0.111%	≤0.111%	>.999
	5	≤0.111%	≤0.111%	>.999
	6	≤0.111%	≤0.111%	>.999
	Total	0.222%	0.245%	.757
▲Total knee arthroplasty	1	0.000%	0.000%	-
	2	≤0.216%	0.000%	.002
	3	≤0.216%	≤0.216%	>.999
	4	≤0.216%	≤0.216%	>.999
	5	≤0.216%	≤0.216%	>.999
	6	≤0.216%	≤0.216%	>.999
	Total	0.315%	0.336%	.857
▲Total hip arthroplasty	1	0.000%	≤0.473%	.002
	2	≤0.473%	≤0.473%	>.999
	3	≤0.473%	≤0.473%	>.999
	4	≤0.473%	≤0.473%	>.999
	5	≤0.473%	≤0.473%	>.999
	6	≤0.473%	≤0.473%	>.999
	Total	0.485%	0.485%	>.999
▲Enlarged tongue	1	≤1.096%	0.000%	.002
	2	≤1.096%	≤1.096%	>.999
	3	≤1.096%	0.000%	.002
	4	0.000%	0.000%	-
	5	≤1.096%	0.000%	.002
	6	0.000%	≤1.096%	.002
	Total	1.143%	1.143%	>.999
▲Dupuytren contracture	1	0.000%	0.000%	-
	2	≤0.284%	≤0.284%	>.999
	3	≤0.284%	≤0.284%	>.999
	4	≤0.284%	≤0.284%	>.999
	5	≤0.284%	≤0.284%	>.999
	6	≤0.284%	≤0.284%	>.999
	Total	0.287%	0.287%	>.999
▲Distal biceps tendon rupture	1	0.000%	≤0.273%	.000
	2	0.000%	≤0.273%	.000
	3	≤0.273%	≤0.273%	>.999
	4	≤0.273%	≤0.273%	>.999
	5	≤0.273%	≤0.273%	>.999
	6	≤0.273%	≤0.273%	>.999
	Total	≤0.273%	0.287%	>.999
▲Pacemaker	1	≤0.449%	0.000%	.000
	2	≤0.449%	0.000%	.000
	3	≤0.449%	0.000%	.000
	4	≤0.449%	0.000%	.000
	5	≤0.449%	≤0.449%	>.999
	6	≤0.449%	≤0.449%	>.999
	Total	0.629%	0.449%	.413

Risk factors shown did not reach statistical significance (*P* < .05) and/or had cohort populations < 5,000 as indicated by a triangle (▲). Risk factors are ordered by descending strength of association.

**Figure 4:**
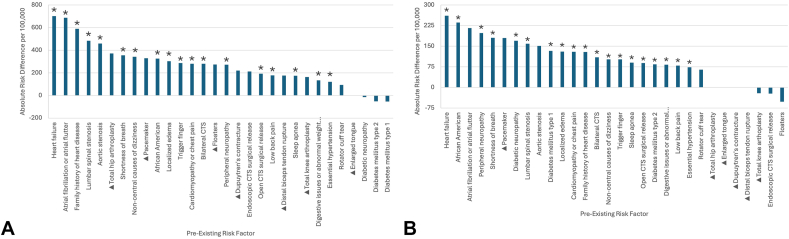
Absolute risk difference per 100,000 of developing amyloidosis within the 6 years following CTS diagnosis. **A** In men aged ≥50. The risk factors are listed strongest to weakest based on absolute risk difference. Asterisks indicate a statistically significant *P* value with *P* < .05. A triangle next to a preexisting risk factor (▲) indicates a cohort population <5,000. **B** In women aged ≥50. The risk factors are listed strongest to weakest based on absolute risk difference. Asterisks indicate a statistically significant *P* value with *P* < .05. A triangle next to a preexisting risk factor (▲) indicates a cohort population <5,000.

**Figure 5:**
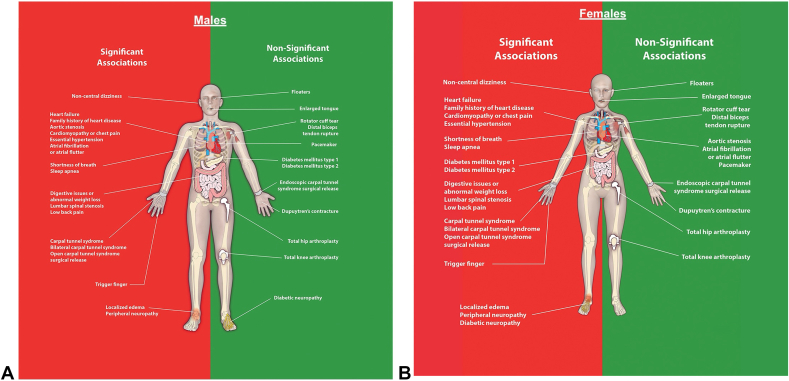
Physical symptoms preceding carpal tunnel diagnosis and their impact on amyloidosis development within 6 years. **A** In men. Significant associations had a *P* < .05. **B** In women. Significant associations had a *P* < .05.

Finally, the preexisting risk factors were ranked from strongest to weakest association based on the risk difference of developing amyloidosis within 6 years following the CTS diagnosis. The results are shown in the Figure 4A for men, with heart failure being the most notable. The ten most notable risk factors for men after heart failure that have an absolute risk difference over 300 per 100,000 are AFib or atrial flutter, family history of heart disease, LSS, AS, THA, shortness of breath, noncentral causes of dizziness, pacemaker, African American race, and localized edema. The results for women are shown in the Figure 4B, with heart failure also being the most considerable. For women, following heart failure, African American race and AFib or atrial flutter were the only other preexisting conditions to have an absolute risk difference over 200 per 100,000. These findings are also presented graphically in the Figure 5A for men and 5B for women.

## Discussion

Previous studies established a link between a subset of patients with CTS who subsequently develop amyloidosis years after their CTS diagnosis.^2^^,^^3^^,^^6^^,^^12^ We retrospectively examined patients with CTS who developed amyloidosis within 6 years of their CTS diagnosis. Patients with ATTRwt, AL, organ-limited, and neuropathic heredofamilial amyloidosis were included, as these subtypes all manifest with cardiac symptoms.^4^ As expected, CTS was more prevalent in women and was most prevalent in 40–60 year-olds.^23^ Similarly, amyloidosis generally affected older individuals and, as expected, impacted men more than women.^16^^,^^24^

The rate of amyloidosis was examined by sex in different ages within the first 6 years of a CTS diagnosis, as prior studies showed that CTS preceded cardiac amyloidosis by 5.1 years.^6^ The youngest age brackets with considerable differences in all 6 years were 50–59-year-old men and 60–69-year-old women. Based on these results, we examined preexisting risk factors before a CTS diagnosis only in patients ≥50 years old, as this cutoff captured patients consistently showing considerable differences in amyloidosis. Furthermore, the population deemed at risk for cardiac amyloidosis is men >65 and women >70, so our age bracket of ≥50 years old includes the most at risk individuals.^22^

Several studies identified “red flag” symptoms linked to a later diagnosis of cardiac amyloidosis. We separated these risk factors by sex, and for men and women, we agree with previous studies’ findings that CTS, LSS, trigger finger, peripheral neuropathy, and bilateral CTS are musculoskeletal “red flags,” but we did not find rotator cuff tear, THA, TKA, or enlarged muscles such as the tongue to be “red flags”.^1^^,^^2^^,^^4^^,^^5^^,^^17^ The men data supported the cardiac “red flags” of heart failure, AFib or atrial flutter, AS, lower-extremity edema, and angina.^12^^,^^16^^,^^18^^,^^19^ The women data supported the same cardiac “red flags,” except for AFib or atrial flutter and AS. As there is no specific ICD-10 code for lower-extremity edema, localized edema was used. Additionally, “angina” was coded as chest pain or cardiomyopathy, as cardiomyopathy can present with chest pain but may not always be coded as such. Impacts on the nervous system can lead to peripheral neuropathy, orthostatic hypotension, and digestive issues, all of which were confirmed “red flags” for both men and women, with orthostatic hypotension included in our “red flag” preexisting condition of noncentral causes of dizziness.^12^^,^^16^ Other potential “red flags” include weight loss, shortness of breath, and sleep apnea. These were also confirmed in men and women.^4^^,^^16^^,^^20^^,^^21^

Hereditary amyloidosis disproportionately impacts African Americans, which is also reflected in both men and women in our data.^25^ We also found men and women with a family history of heart disease and low back pain, which can be a symptom of LSS, to be “red flags” for cardiac amyloidosis.^26^ Additional “red flags” identified for women involved essential hypertension, diabetes mellitus type 1, diabetes mellitus type 2, and diabetic neuropathy.

Our study built upon a study that tiered risk factors to help guide hand surgeons’ decisions on tenosynovial biopsies. The previous study placed demographics and bilateral CTS or CTS surgical release in one tier and six other comorbidities in another tier to form a diagnostic algorithm.^12^ This tiering system has been taken a step further by individually examining comorbidities and patient characteristics and ranking them based on their clinical and statistical significance. We agree that men ≥50 should be included, and women should also be included at ≥50 rather than ≥60. Bilateral CTS is a risk factor, but should be moved to tier two, and heart failure should be moved to tier one, as it was the strongest “red flag”. For tier two, we agree that spinal stenosis, specifically lumbar, for both men and women, and AFib or atrial flutter for men, should be included. Limited by ICD-10 codes, we could not examine how a family history of ATTR amyloidosis may impact a patient’s risk.

Recently, Ruesch et al^11^ developed a similar algorithm to identify patients with CTS at risk for cardiac amyloidosis, which included the criteria of age (men ≥50, women ≥60), CTS, spinal stenosis, biceps tendon rupture, AFib or flutter, pacemaker, CHF, and family history of ATTR.^11^ They evaluated these data and performed Congo red staining of interoperative tissues (transverse carpal ligament or flexor tendon sheath) to identify patients at risk for cardiac ATTR. They identified six patients with positive Congo red staining, and one was started on cardiac disease-modifying treatment. While this study supports the value of “red flags,” it did not justify routine biopsies and concluded that neither biopsies nor early disease-modifying treatment is currently cost effective.

While identifying several notable preexisting risk factors, it is important to note the scale of these associations. For the most notable risk factor of heart failure, only 700 of 100,000 men with CTS and heart failure developed amyloidosis within 6 years. For the remaining risk factors, the number of patients was even smaller. For that reason, it is essential that this information be refined to create a clinically practical diagnostic screening tool.

Our study has limitations. Using ICD-10 and CPT codes to define patient characteristics and comorbidities may introduce some misclassification of patients. Moreover, amyloidosis is a rare disease, and billing codes may underestimate the number of patients with amyloidosis. We also cannot consider who is diagnosing CTS and based on what criteria. Additionally, we are limited in the diagnoses we can study based on the available ICD-10 and CPT codes and the HCOs present in TriNetX’s network of 144 million patients.

Despite these limitations, our large cohort study assessed preexisting risk factors for CTS patients who develop amyloidosis. We suggest that these data be used to develop a weighted tool to identify patients with CTS who should have a tenosynovial biopsy at surgery. While the number of patients at risk for amyloidosis is small, it is critical to identify this cohort of patients early to prevent fatal myocardial disease. New medications can slow the progression of amyloidosis, and these drugs are most effective in the earlier stages of the disease.^12^^,^^14^ Therefore, hand surgeons can play a critical role in identifying patients at risk for amyloidosis so that early intervention can prolong their lives.

## Conflicts of Interest

No benefits in any form have been received or will be received related directly to this article.
